# Leukocytes Do Not Influence the Safety and Efficacy of Platelet-Rich Plasma Injections for the Treatment of Knee Osteoarthritis: A Double-Blind Randomized Controlled Trial

**DOI:** 10.1177/03635465241283500

**Published:** 2024-10-12

**Authors:** Iacopo Romandini, Angelo Boffa, Alessandro Di Martino, Luca Andriolo, Annarita Cenacchi, Elena Sangiorgi, Simone Orazi, Valeria Pizzuti, Stefano Zaffagnini, Giuseppe Filardo

**Affiliations:** * Clinica Ortopedica e Traumatologica II, IRCCS Istituto Ortopedico Rizzoli, Bologna, Italy; ‡ Servizio Trasfusionale Unico Metropolitano, Bologna, Italy; § Applied and Translational Research (ATR) Center, IRCCS Istituto Ortopedico Rizzoli, Bologna, Italy; ∥ Faculty of Biomedical Sciences, Università della Svizzera Italiana, Lugano, Switzerland; Investigation performed at IRCCS Istituto Ortopedico Rizzoli, Bologna, Italy

**Keywords:** knee, osteoarthritis, platelet-rich plasma (PRP), leukocytes, injection, intra-articular

## Abstract

**Background:**

Platelet-rich plasma (PRP) is increasingly used for the injection treatment of knee osteoarthritis (OA). However, the role of leukocytes contained in PRP is controversial, with some preclinical studies suggesting detrimental effects and others emphasizing their contribution in secreting bioactive molecules.

**Purpose:**

To compare the safety and effectiveness of leukocyte-rich PRP (LR-PRP) and leukocyte-poor PRP (LP-PRP) for the treatment of knee OA.

**Hypothesis:**

That leukocytes could influence results both in terms of adverse events and clinical outcomes.

**Study Design:**

Randomized controlled trial; Level of evidence, 1.

**Methods:**

This double-blind randomized controlled trial included 132 patients with Kellgren-Lawrence grade 1-3 knee OA who were randomized to a 3-injection cycle of either LR-PRP or LP-PRP. Patients were prospectively assessed at baseline and at 2, 6, and 12 months with subjective evaluations comprising the International Knee Documentation Committee (IKDC) subjective score, the KOOS (Knee injury and Osteoarthritis Outcome Score), the WOMAC (Western Ontario and McMaster Universities Osteoarthritis Index), the visual analog scale for pain, the EuroQol–visual analog scale, the EuroQol–5 dimensions, and the Tegner activity scale. Objective evaluations consisted of the IKDC objective score, active/passive range of motion, and circumference of the index and contralateral knees. Patient judgment of the treatment was recorded as well as adverse reactions and failures.

**Results:**

An overall improvement in subjective and objective outcomes was documented, with no differences between the 2 groups, except for the improvement in the IKDC subjective score at 2 months, which was greater for the LR-PRP group compared with the LP-PRP group (14.8 ± 14.8 vs 8.6 ± 13.3, respectively; *P* = .046), as well as for active (*P* = .021) and passive (*P* = .040) ROM of the index knee at 6 months, showing statistically significant higher values in the LP-PRP group; and for quadriceps circumference of the index (*P* = .042) and contralateral (*P* = .045) knees at 12 months, which were significantly greater in the LR-PRP group. The IKDC subjective score improved from 42.5 ± 17.6 at baseline to 55.6 ± 21.4 at 12 months for the LR-PRP group (*P* < .0005) and from 45.7 ± 16.4 to 55.3 ± 20.4 for the LP-PRP group (*P* = .001). No differences in terms of patient treatment judgment were observed at all follow-up time points. No severe adverse events related to the treatment were reported, but some mild adverse events related to the treatment were observed: 16 in the LR-PRP group and 17 in the LP-PRP group. Treatment failed in 5 patients in the LR-PRP group and 2 in the LP-PRP group.

**Conclusion:**

This double-blind randomized controlled trial demonstrated that leukocytes did not affect the safety and efficacy of intra-articular PRP injections for the treatment of patients with knee OA. Both LR-PRP and LP-PRP demonstrated comparable clinical outcomes at all follow-up time points, without showing differences in subjective and objective outcomes or in adverse events and treatment failures.

**Registration:**

NCT04187183 (ClinicalTrials.gov).

Knee osteoarthritis (OA) is a degenerative joint condition that leads to the progressive deterioration and loss of articular cartilage, with concomitant structural and functional changes in the entire joint.^23,30^ This disorder is one of the primary causes of debilitating pain, disability, and physical limitations in adults, bearing a significant effect both socially and economically.^30,31^ Various therapeutic options can be used to address knee OA, ranging from nonoperative treatment to invasive surgical interventions. Joint replacement is effective in addressing the severe forms of OA in the elderly,^33^ but its use is questionable in younger patients in the early stages of the disease,^12,25^ who can benefit instead from a nonoperative approach including physical therapy, weight loss, and pharmacological drugs to alleviate symptoms and preserve joint function.^22,28,30^ When nonoperative treatment fails, injection therapies are commonly adopted, with corticosteroids and hyaluronic acid representing the most common choices. However, these injections may improve symptoms in the short term but exhibit limited long-term efficacy.^4,26^ This limitation has stimulated research into new products capable of providing enduring clinical benefits and possibly altering the disease progression.^11^

Platelet-rich plasma (PRP) injection has emerged in this context as a promising alternative for the treatment of knee OA.^3^ PRP injections have demonstrated safety, ease of preparation, and disease-modifying effects in OA animal models, attenuating the progression of cartilage tissue damage and reducing inflammatory reactions of the synovial membrane.^7^ Several clinical studies have shown satisfactory results in terms of functional and symptom improvement after PRP injections, with systematic reviews and meta-analyses confirming better results with PRP compared with saline and other injectable options, such as corticosteroids and hyaluronic acid.^15,24,29,32^ However, some controversial aspects persist in the use of PRP injections. Among these, the role of leukocytes contained in PRP represents one of the most debated aspects, and PRP formulations are generally divided into 2 main categories based on the leukocyte concentration: leukocyte-poor PRP (LP-PRP) and leukocyte-rich PRP (LR-PRP).^1^ While some preclinical studies have suggested detrimental effects associated with the presence of leukocytes,^8^ others have emphasized their benefit in controlling joint inflammation.^16^ Nevertheless, evidence on the effects of leukocytes in the clinical setting remains limited.

The primary aim of this double-blind randomized controlled trial (RCT) comparing LP-PRP versus LR-PRP injections was to investigate if leukocytes affect the efficacy of PRP injections for the treatment of knee OA. The secondary aim was to explore if the presence of leukocytes can influence the safety of PRP injections. The hypothesis was that leukocytes could influence results both in terms of adverse events and clinical outcomes.

## Methods

### Patient Selection

This double-blind RCT, registered on ClinicalTrials.gov (NCT04187183), was entirely conducted at IRCCS Istituto Ortopedico Rizzoli and approved by the hospital's ethics committee (No. 0013413). Patients were recruited by orthopaedic physicians between June 2020 and August 2022, and informed consent was obtained from each patient for study participation. The following inclusion criteria were selected: patients with symptomatic unilateral knee OA (at least 6 months of chronic pain or swelling), age 18-75 years, radiographic or magnetic resonance imaging signs of degenerative abnormalities of knee cartilage (Kellgren-Lawrence grade 1-3), and unsatisfactory outcomes after at least 6 months of nonoperative treatment (rest, physical therapy, anti-inflammatory and analgesic medications, and reduction of physical activities). The following exclusion criteria were selected: bilateral symptoms, history of trauma or intra-articular injections within 6 months before treatment or knee surgery within 12 months, significant lower limb malalignment (varus >5°, valgus >5°), presence of concomitant symptomatic knee lesions requiring surgery (eg, untreated knee instability, meniscal injuries, focal chondral or osteochondral lesions), malignant neoplastic diseases, systemic disorders (eg, uncontrolled diabetes), uncontrolled thyroid metabolic disorders, severe cardiovascular disease, rheumatoid arthritis, inflammatory arthropathy, hematological disorders, infections, immunosuppression, anticoagulant or antiplatelet therapy, use of nonsteroidal anti-inflammatory drugs within 5 days before blood harvesting, hemoglobin level <11 g/dL, or platelet count <150,000/mm^3^.

### Randomization and Blinding

After the enrollment of patients into the study, they were randomly allocated to 2 treatment groups: one received 3 weekly intra-articular injections of LR-PRP, while the other received 3 weekly intra-articular injections of LP-PRP. The allocation sequence was generated by an independent statistician using a random number generator and then stored in a dedicated armored cabinet. Treatment assignments were progressively numbered within sealed envelopes. A clinical monitor used these envelopes to inform the hematologist about the treatment to be administered. Patients, physicians administering intra-articular therapy, and clinical investigators assessing the patients at all follow-up visits were all blinded to the type of PRP used (only the hematologists responsible for preparing the 2 types of PRP were aware of the study groups). Both LR-PRP and LP-PRP preparations shared identical macroscopic characteristics, making it impossible for patients and administering physicians to differentiate them. Data provided to the statistician were coded with generic labels (group A and group B), concealing the specific intervention associated with each group. This approach ensured that the statistician remained unaware of treatment allocations throughout the analysis process. The specific group assignment was communicated to the patient only after completion of the study.

### PRP Preparation and Administration Protocol

Both PRP preparations were produced using the CPunT system (Eltek Group). The CPunT system is a device that induces the separation of PRP from anticoagulated whole blood in a closed system, consisting of a medical electrical machine, one disposable, and the centrifuge. In a sterile manner, 50 mL of whole blood was collected from each patient into a 60-mL syringe with 7 mL of anticoagulant citrate dextrose solution. A first centrifugation at 1200 rpm for 10 minutes was performed to separate blood into platelet-poor plasma, the buffy coat, and red blood cells (RBCs). The disposable was then placed in the machine, which pushed plasma and platelets out of the syringe and into a bag, which contained the final product. The machine was designed to end the separation cycle when its sensor detected RBCs entering the bag and had an additional optional setting (only for the LR-PRP arm), which allowed for the collection of leukocytes in this phase by recovering an additional fixed volume of fluid. The syringe containing RBCs was discarded, and the bag underwent a second centrifugation at 1900 rpm for 10 minutes, which concentrated the platelets and leukocytes if present. Excess plasma was removed with a syringe, with the goal of concentrating the platelets at 1 × 10^6^/μL ± 20%. After disaggregation of the platelets via manual manipulation and mechanical agitation, the final product (5 mL) was collected with a syringe and was ready for use. When the leukocyte retrieval option was selected during separation, the product contained a higher concentration of white blood cells compared with the patient's blood level. Conversely, when the leukocytes were not harvested, the final concentration was lower compared with the patient's blood level.

The PRP sample was directly transferred from the transfusion unit to the outpatient clinic within the hospital, using a thermal bag to shield it from light exposure. Patients in both groups received treatment by orthopaedic surgeons specializing in injection therapies. The treatment plan involved 3 injections at 1-week intervals. Before each injection, PRP was activated by introducing 0.5 mL of calcium gluconate. The injection site was sterilized with antiseptic solution, and the injection was administered using a 22-gauge needle through a superolateral parapatellar approach. Upon completion of the procedure, patients were encouraged to flex and extend their knee multiple times to distribute PRP throughout the entire joint.

After the injection, patients were discharged with instructions to limit the use of the treated limb (avoiding vigorous sports or activities involving the treated knee) for at least 24 to 48 hours and were advised to apply ice to the area to reduce possible pain or swelling. Throughout the injection cycle, rest combined with light activities was recommended, without imposing restrictions on knee weightbearing or range of motion (ROM), allowing for a gradual return to normal sports or recreational activities based on individual tolerance. The use of oral anti-inflammatory drugs was discouraged, particularly in the 5 days before each follow-up visit.

### Patient Evaluation

The characteristics of the patients were collected, including sex, age, body mass index, side of the index knee, symptom duration, previous injections and surgery, and OA grade according to the Kellgren-Lawrence classification. All patients were prospectively assessed at baseline and at 2, 6, and 12 months after the last injection. To ensure double blinding of the trial, all clinical subjective and objective evaluations were conducted by independent physicians who were not involved in the injection procedure and were blinded to the randomization list. Clinical scores were collected through paper questionnaires during visits to the outpatient clinic, with patients filling out the questionnaires and physicians being available for clarification. The primary clinical outcome was the change in the International Knee Documentation Committee (IKDC) subjective score at 12 months after the injections. The following secondary subjective scores were collected at baseline and at each follow-up visit: the IKDC subjective score, the Knee injury and Osteoarthritis Outcome Score (KOOS) and the Western Ontario and McMaster Universities Osteoarthritis Index (WOMAC) for function, the visual analog scale (VAS) for pain, the EuroQol–visual analog scale (EQ-VAS) for health perception, the EuroQol–5 dimensions (EQ-5D) for health-related quality of life, and the Tegner activity scale for sport/activity level. Additionally, objective data were recorded to assess patients at baseline and at each follow-up visit: the IKDC objective score, active and passive ROM of the index and contralateral knees, circumference of the index and contralateral knees (measured at the center of the patella), and quadriceps circumference of the index and contralateral knees (measured at 10 cm from the center of the patella). Finally, patient judgment of the treatment was examined at 2, 6, and 12 months using a specific question: “Compared to the initial state, how would you rate the treated knee now?” Responses were recorded using a 5-point scale: “much better,”“somewhat better,”“about the same,”“somewhat worse,” and “much worse.”

Any complications and adverse reactions were documented and managed after each injection and during every follow-up visit, ensuring the safety of both PRP preparations. Mild adverse events were defined as the persistence of significant knee pain or swelling in the treated knee for >5 days, as reported by patients. Severe adverse events were identified as any situation requiring hospitalization or interventions to prevent permanent damage. Patients who required new injections or surgical interventions because of persistent or worsening symptoms were considered as treatment failures. In these cases, the worst clinical evaluation between baseline and the last available follow-up was considered for subsequent follow-up.

### Statistical Analysis

The sample size was determined by performing a power analysis on the primary outcome (change in the IKDC subjective score at 12-month follow-up). A previous pilot study revealed a standard deviation of 16.7 points. With an alpha of .05 and a power of 0.90, and a clinically significant difference of 10 points for the IKDC, the minimum sample size required was 60 patients per group, for a total of 120 patients. Considering a 10% potential dropout rate, 66 patients were needed per group, for a total of 132 patients. All continuous data were expressed as the mean and standard deviation, and categorical data were expressed as the frequency and percentage. The Shapiro-Wilk test was used to assess the normality of continuous variables. The repeated-measures general linear model with the Sidak test for multiple comparisons was used to assess differences in quantitative outcomes at different follow-up time points. The Friedman nonparametric test, followed by the Wilcoxon pairwise test with the Bonferroni correction, was used to assess differences in ordinal outcomes at different follow-up time points. One-way analysis of variance with the Scheffé post hoc pairwise test was used to assess differences between groups when the Levene test for the homogeneity of variance was not significant (*P* < .05); otherwise, the Mann-Whitney test (2 groups) or the Kruskal-Wallis test with the Dunnett nonparametric post hoc pairwise test was used. The Spearman rank correlation coefficient was used to assess correlations between quantitative outcomes and continuous data. The Kendall tau correlation coefficient was used to assess correlations between quantitative or ordinal outcomes and categorical data. The Fisher exact test was used to assess the relationship between dichotomous variables. The chi-square test was used to investigate relationships between categorical variables. Kaplan-Meier survival analysis was performed to estimate the failure rate; the log-rank test was used to assess the influence of the treatment on survival. For all tests, *P* < .05 was considered significant. All statistical analyses were performed using SPSS (Version 19.0; IBM).

## Results

### Patient Characteristics

**Figure 1.**
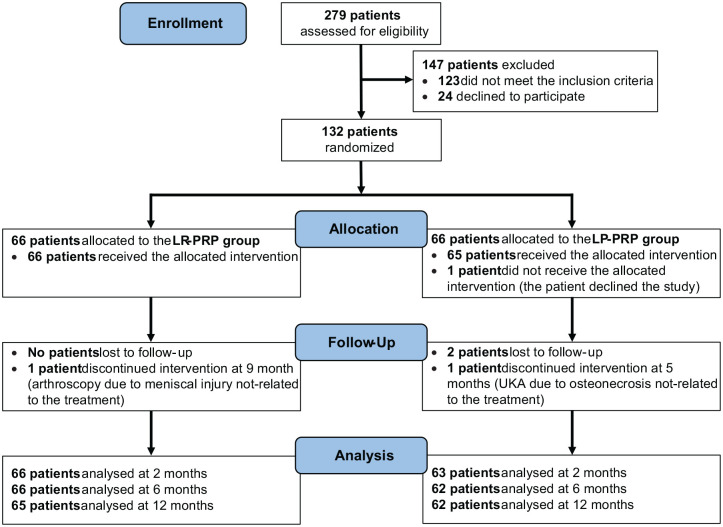
CONSORT (Consolidated Standards of Reporting Trials) flow diagram showing patient inclusion and follow-up. LP-PRP, leukocyte-poor platelet-rich plasma; LR-PRP, leukocyte-rich platelet-rich plasma; UKA, unicompartmental knee arthroplasty.

A total of 132 patients met the inclusion criteria and were randomized (Figure 1). One patient did not receive the allocated intervention after being enrolled (declined to participate after allocation), while 2 patients were considered as dropouts because of unavailability (for personal reasons) during follow-up visits and were excluded from the analysis. Therefore, the study population consisted of 129 patients: 66 in the LR-PRP group and 63 in the LP-PRP group.

The 2 groups were homogeneous for all baseline characteristics, except for age (*P* = .044), EQ-VAS score (*P* = .041), and active and passive ROM of the index knee (*P* = .007 and *P* = .003, respectively). The baseline characteristics and clinical scores of the 2 groups are reported in Tables 1, 4, and 5.

**Table 1:** Baseline Patient Characteristics^*a*^

	LR-PRP (n = 66)	LP-PRP (n = 63)
Sex		
Female	29	35
Male	37	28
Age, y	56.7 ± 10.2	51.9 ± 13.5
Side		
Right	37	28
Left	29	35
Body mass index	25.2 ± 3.8	24.4 ± 3.4
Smoking		
No	51	53
Yes	15	10
Symptom duration, mo	52.2 ± 48.7	66.7 ± 87.6
Kellgren-Lawrence classification		
Grade 1	15	14
Grade 2	33	33
Grade 3	18	16
Previous injections		
No	16	13
Yes	50	50
Previous surgery		
No	34	29
Yes	32	34

a Data are expressed as mean ± SD or No. LP-PRP, leukocyte-poor platelet-rich plasma; LR-PRP, leukocyte-rich platelet-rich plasma.

### PRP Characteristics

The number of platelets per milliliter increased after the centrifugation process by a mean of 4.3 times in the LR-PRP group and 3.7 times in the LP-PRP group with respect to baseline whole blood values, with no significant difference between the 2 groups (not significant). The leukocyte concentration was significantly higher in the LR-PRP group than in the LP-PRP group (*P* < .0005). Leukocytes were present with a mean concentration of 2.3 times with respect to the whole blood value in the LR-PRP group, while the mean concentration was 0.8 times compared with whole blood values in the LP-PRP group. A significant difference in the concentration of remaining RBCs after PRP preparation between the 2 groups was also observed (*P* < .0005). Further details on the 2 PRP formulations are reported in Tables 2 and 3.

**Table 2:** Concentrations of Platelets, Leukocytes, and Erythrocytes in Whole Blood and PRP^*a*^

	LR-PRP (n = 66)	LP-PRP (n = 63)	*P* Value
Platelets, ×10^9^/L			
Whole blood	236.7 ± 33.6	251.8 ± 39.7	.028
PRP	1009.3 ± 213.9	939.3 ± 190.3	NS
Leukocytes, ×10^6^/L			
Whole blood	6.4 ± 1.4	6.0 ± 1.5	NS
PRP	14.4 ± 4.9	4.7 ± 2.4	<.0005
Erythrocytes, ×10^9^/L			
Whole blood	4.9 ± 1.2	4.8 ± 0.4	NS
PRP	0.9 ± 0.2	0.1 ± 0.1	<.0005

a Data are expressed as mean ± SD. LP-PRP, leukocyte-poor platelet-rich plasma; LR-PRP, leukocyte-rich platelet-rich plasma; NS, not significant.

**Table 3:** Leukocyte Concentrations^*a*^

	LR-PRP (n = 66)	LP-PRP (n = 63)	*P* Value
Neutrophils, ×10^6^/L	5.9 ± 4.2	0.7 ± 0.8	<.0005
Lymphocytes, ×10^6^/L	8.1 ± 4.1	4.3 ± 5.0	<.0005
Monocytes, ×10^6^/L	1.7 ± 0.8	0.6 ± 0.8	<.0005
Eosinophils, ×10^6^/L	0.1 ± 0.1	0.0 ± 0.0	<.0005
Basophils, ×10^6^/L	0.0 ± 0.0	0.0 ± 0.0	<.0005

a Data are expressed as mean ± SD. LP-PRP, leukocyte-poor platelet-rich plasma; LR-PRP, leukocyte-rich platelet-rich plasma.

### Adverse Events and Failures

No severe adverse events related to the treatment were reported in either group, but some mild adverse events related to the treatment were observed: 16 (24.2%) in the LR-PRP group and 17 (27.0%) in the LP-PRP group, such as knee pain and swelling for >5 days after the injection, which resolved within a few days with rest, ice, and pain-relieving medications (acetaminophen), with no statistically significant differences between the 2 groups (not significant). Another 2 patients experienced adverse events unrelated to the treatment: in the LR-PRP group, 1 patient underwent knee arthroscopic surgery because of a meniscal tear after a traumatic injury at 9 months after the treatment and was excluded from the final analysis; in the LP-PRP group, 1 patient was diagnosed with osteonecrosis of the medial femoral condyle during the course of the investigation and underwent medial unicompartmental knee arthroplasty at 5 months after the treatment. These 2 patients were excluded from the 6- and 12-month analyses. Regarding treatment failures, 5 (7.6%) patients had failure in the LR-PRP group. Three patients received corticosteroid injections for persistent pain and swelling at 2, 3, and 11 months, respectively, after the treatment. One patient received a hyaluronic acid injection at 4 months, while another patient underwent knee arthroscopic surgery, revealing degenerative meniscopathy and chronic hypertrophic synovitis at 11 months after the treatment. In the LP-PRP group, 2 (3.2%) patients were considered treatment failures: 1 patient received a corticosteroid injection at 10 months for persistent pain and swelling, and another patient received a hyaluronic acid injection at 10 months after the treatment. No statistically significant difference was found between the LR-PRP and LP-PRP groups in terms of survival rates at the final follow-up (92.4% vs 96.8%, respectively; not significant).

### Subjective and Objective Clinical Outcomes

**Figure 2.**
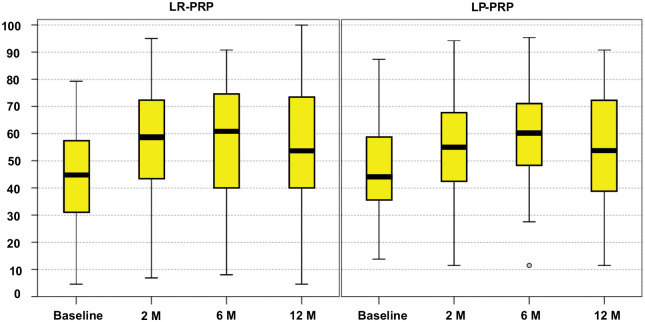
International Knee Documentation Committee subjective score in both treatment groups at baseline and at 2-, 6-, and 12-month follow-up. The box and whisker plots show the median, interquartile range, and range. LP-PRP, leukocyte-poor platelet-rich plasma; LR-PRP, leukocyte-rich platelet-rich plasma.

An overall improvement in subjective clinical scores was documented for both groups from baseline to each follow-up time point (Table 4). The mean IKDC subjective score (Figure 2) significantly improved at 12 months, increasing from 42.5 ± 17.6 to 55.6 ± 21.4 for the LR-PRP group (*P* < .0005) and from 45.7 ± 16.4 to 55.3 ± 20.4 for the LP-PRP group (*P* = .001). Both groups reached the minimal clinically important difference^6^ for the primary outcome (change in the IKDC subjective score from baseline to 12 months). The comparative analysis of the primary outcome did not show a significant difference between groups (not significant). Both groups reported a comparable treatment judgment, with the knee rated as “improved” in 48.5% (42.4% as “much better” and 6.1% as “somewhat better”) of the LR-PRP group and in 44.5% (41.3% as “much better” and 3.2% as “somewhat better”) of the LP-PRP group at 2 months (not significant), in 48.5% (39.4% as “much better” and 9.1% as “somewhat better”) of the LR-PRP group and in 51.6% (46.8% as “much better” and 4.8% as “somewhat better”) of the LP-PRP group at 6 months (not significant), and in 52.3% (38.5% as “much better” and 13.8% as “somewhat better”) of the LR-PRP group and in 48.4% (45.2% as “much better” and 3.2% as “somewhat better”) of the LP-PRP group at 12 months (not significant). Additionally, no differences between the 2 groups were observed in terms of absolute values and improvement in other clinical subjective scores, except for the improvement in the IKDC subjective score from baseline to 2 months of follow-up, which showed a significantly greater value (*P* = .046) in the LR-PRP group (14.8 ± 14.8) compared with the LP-PRP group (8.6 ± 13.3). All subjective outcomes at baseline and at 2, 6, and 12 months are reported in Table 4.

**Table 4:** Subjective Outcomes^*a*^

	Baseline	2 mo	6 mo	12 mo	*P* Value (ANOVA)
IKDC subjective					
LR-PRP	42.5 ± 17.6	57.2 ± 17.7	57.3 ± 20.7	55.6 ± 21.4	<.0005
LP-PRP	45.7 ± 16.4	54.8 ± 18.2	58.5 ± 19.4	55.3 ± 20.4	<.0005
VAS pain					
LR-PRP	59.4 ± 22.7	42.9 ± 24.9	41.2 ± 26.4	43.9 ± 26.3	<.0005
LP-PRP	57.9 ± 22.1	43.1 ± 26.0	41.0 ± 23.9	44.7 ± 24.9	<.0005
EQ-VAS					
LR-PRP	66.2 ± 18.1^*b*^	75.5 ± 13.9	75.4 ± 15.2	72.0 ± 18.4	<.0005
LP-PRP	73.0 ± 14.7^*b*^	73.8 ± 17.1	76.2 ± 14.4	75.5 ± 15.1	NS
EQ-5D					
LR-PRP	0.6 ± 0.3	0.7 ± 0.3	0.8 ± 0.3	0.7 ± 0.3	<.0005
LP-PRP	0.6 ± 0.3	0.7 ± 0.2	0.8 ± 0.2	0.7 ± 0.3	<.0005
WOMAC Pain					
LR-PRP	6.0 ± 4.0	3.6 ± 3.6	3.4 ± 3.3	3.9 ± 4.2	<.0005
LP-PRP	5.1 ± 3.7	3.2 ± 3.1	2.8 ± 2.7	3.9 ± 3.8	<.0005
WOMAC Stiffness					
LR-PRP	3.0 ± 1.9	2.0 ± 1.8	1.8 ± 1.8	2.0 ± 2.0	<.0005
LP-PRP	2.6 ± 1.8	2.0 ± 1.7	1.9 ± 1.7	2.2 ± 2.0	.015
WOMAC Function					
LR-PRP	21.9 ± 14.6	14.2 ± 14.4	13.5 ± 13.3	15.5 ± 15.8	<.0005
LP-PRP	19.0 ± 13.3	13.7 ± 11.8	12.5 ± 12.2	15.4 ± 13.5	.001
WOMAC Total					
LR-PRP	30.9 ± 19.9	19.7 ± 19.4	18.7 ± 18.1	21.4 ± 21.5	<.0005
LP-PRP	26.6 ± 17.9	18.9 ± 15.9	17.2 ± 15.9	21.6 ± 18.6	<.0005
KOOS Symptoms					
LR-PRP	62.2 ± 19.4	70.3 ± 18.9	72.1 ± 21.1	72.8 ± 22.0	<.0005
LP-PRP	61.8 ± 17.9	71.1 ± 17.2	71.5 ± 19.3	69.6 ± 18.9	<.0005
KOOS Pain					
LR-PRP	61.8 ± 19.0	73.8 ± 19.4	74.9 ± 20.0	72.7 ± 21.4	<.0005
LP-PRP	65.7 ± 18.4	74.7 ± 19.2	76.5 ± 19.0	74.5 ± 20.2	.001
KOOS Sport					
LR-PRP	47.6 ± 20.0	56.9 ± 22.4	57.7 ± 24.4	56.5 ± 26.8	.001
LP-PRP	46.3 ± 19.4	55.0 ± 23.4	59.8 ± 24.7	57.3 ± 25.1	.001
KOOS Activities of Daily Living					
LR-PRP	69.7 ± 21.1	81.3 ± 19.3	81.1 ± 20.1	78.1 ± 22.4	<.0005
LP-PRP	74.4 ± 18.9	81.8 ± 17.8	83.8 ± 17.7	80.4 ± 20.5	<.0005
KOOS Quality of Life					
LR-PRP	36.7 ± 19.3	50.1 ± 23.5	54.2 ± 24.6	51.6 ± 27.1	<.0005
LP-PRP	36.7 ± 19.1	49.5 ± 23.1	53.2 ± 23.2	51.5 ± 25.0	<.0005
Tegner					
LR-PRP	2.4 ± 1.4	3.0 ± 1.4	2.8 ± 1.3	3.0 ± 1.7	.014
LP-PRP	2.4 ± 1.3	2.6 ± 1.3	3.1 ± 1.6	3.1 ± 1.7	.003

a Data are expressed as mean ± SD. ANOVA, analysis of variance; EQ-5D, EuroQol–5 dimensions; EQ-VAS, EuroQol–visual analog scale; IKDC, International Knee Documentation Committee; KOOS, Knee injury and Osteoarthritis Outcome Score; LP-PRP, leukocyte-poor platelet-rich plasma; LR-PRP, leukocyte-rich platelet-rich plasma; NS, not significant; VAS, visual analog scale; WOMAC, Western Ontario and McMaster Universities Osteoarthritis Index.

b *P* < .05 in favor of the LP-PRP group.

An overall improvement was also reported in objective clinical outcomes from baseline to each follow-up time point for both groups (Table 5). The IKDC objective score significantly improved from baseline to the final follow-up in both the LR-PRP group (from 2.8 ± 0.7 to 2.2 ± 1.0; *P* < .0005) and the LP-PRP group (from 2.7 ± 0.9 to 2.0 ± 0.7; *P* < .0005). No differences between the 2 groups were observed in terms of absolute values and improvement in objective outcomes, except for active (*P* = .021) and passive (*P* = .040) ROM of the index knee at 6 months, showing statistically significant higher values in the LP-PRP group, and except for quadriceps circumference of the index (*P* = .042) and contralateral (*P* = .045) knees at 12 months, which were significantly greater in the LR-PRP group. All objective outcomes at baseline and at 2, 6, and 12 months are reported in Table 5.

**Table 5:** Objective Outcomes^*a*^

	Baseline	2 mo	6 mo	12 mo	*P* Value (ANOVA)
IKDC objective					
LR-PRP	2.8 ± 0.7	2.3 ± 0.8	2.3 ± 1.0	2.2 ± 1.0	<.0005
LP-PRP	2.7 ± 0.9	2.1 ± 0.8	2.2 ± 0.9	2.0 ± 0.7	<.0005
ROM active index knee					
LR-PRP	120.1 ± 11.4^*b*^	125.2 ± 11.0	124.3 ± 11.7^*b*^	125.3 ± 12.5	<.0005
LP-PRP	125.7 ± 12.7^*b*^	128.4 ± 10.6	128.7 ± 9.2^*b*^	127.7 ± 9.3	NS
ROM active contralateral knee					
LR-PRP	132.1 ± 9.2	132.9 ± 8.7	133.6 ± 9.7	133.1 ± 9.1	NS
LP-PRP	133.8 ± 15.4	134.8 ± 9.8	133.5 ± 9.5	133.2 ± 6.9	NS
ROM passive index knee					
LR-PRP	124.5 ± 10.7^*b*^	129.7 ± 10.7	129.2 ± 11.9^*b*^	130.1 ± 12.6	<.0005
LP-PRP	130.3 ± 12.9^*b*^	132.3 ± 10.4	133.0 ± 8.4^*b*^	131.9 ± 9.2	NS
ROM passive contralateral knee					
LR-PRP	127.7 ± 9.3	128.3 ± 8.9	128.9 ± 9.5	128.1 ± 8.9	NS
LP-PRP	130.8 ± 8.8	130.7 ± 10.4	129.1 ± 10.1	129.1 ± 7.2	NS
Circumference index knee					
LR-PRP	381.6 ± 28.6	382.7 ± 30.3	384.3 ± 27.6	387.1 ± 31.2	NS
LP-PRP	375.6 ± 30.5	373.7 ± 31.3	378.8 ± 34.6	378.3 ± 34.0	NS
Circumference contralateral knee					
LR-PRP	379.6 ± 25.3	381.4 ± 27.3	380.1 ± 25.3	383.3 ± 28.8	NS
LP-PRP	371.7 ± 31.8	370.6 ± 32.6	375.8 ± 34.2	376.9 ± 36.3	NS
Quadriceps circumference index knee					
LR-PRP	425.7 ± 39.9	424.7 ± 39.5	433.6 ± 44.7	440.8 ± 43.4^*c*^	<.0005
LP-PRP	425.4 ± 42.7	425.6 ± 44.0	428.2 ± 48.1	424.2 ± 45.6^*c*^	NS
Quadriceps circumference contralateral knee					
LR-PRP	428.0 ± 40.4	427.8 ± 40.9	437.7 ± 44.0	441.5 ± 45.5^*c*^	.003
LP-PRP	426.2 ± 42.5	429.6 ± 43.2	427.6 ± 47.3	426.4 ± 45.7^*c*^	NS

a Data are expressed as mean ± SD. ANOVA, analysis of variance; IKDC, International Knee Documentation Committee; LP-PRP, leukocyte-poor platelet-rich plasma; LR-PRP, leukocyte-rich platelet-rich plasma; NS, not significant; ROM, range of motion.

b *P* < .05 in favor of the LP-PRP group.

c *P* < .05 in favor of the LR-PRP group.

## Discussion

The main finding of this double-blind RCT is that leukocytes did not affect the safety and efficacy of intra-articular PRP injections for the treatment of patients with knee OA. Knees treated with LR-PRP and LP-PRP demonstrated comparable clinical outcomes at all follow-up time points, without showing differences in subjective and objective outcomes or in adverse events and treatment failures.

The presence of leukocytes is the most debated aspect of PRP injections in both scientific research and the clinical setting. Several studies have suggested a pro-inflammatory role played by leukocytes both in preclinical models and the clinical setting. In vitro experiments have documented that leukocytes may alter PRP properties by releasing catabolic and pro-inflammatory molecules, potentially harming joint tissue.^2,10^ Similarly, in vivo studies have confirmed the pro-inflammatory properties of LR-PRP in animal models of OA, documenting increased pro-inflammatory biomarkers, such as interleukin-1β (IL-1β) and prostaglandin E2, after LR-PRP treatment compared with LP-PRP, thus supporting the possible negative effect of leukocytes when administered into the joint. The potential detrimental effects of leukocytes have also been suggested in the clinical setting in which preliminary studies reported higher rates of pain and swelling after injections of LR-PRP compared with LP-PRP.^14^ Moreover, a cytokine analysis of PRP with and without leukocytes obtained from the same patients documented a higher expression of IL-4, IL-8, and matrix metalloproteinase-9 (MMP-9) in LR-PRP compared with LP-PRP.^17^ Nevertheless, a biomarker analysis after LR-PRP injections did not confirm pro-inflammatory effects in human joints, yielding inconsistent findings compared with animal model findings. In a study on 36 patients with knee OA, an evaluation of pro-inflammatory cytokine levels in synovial fluid and plasma at 1 week after an intra-articular LR-PRP injection did not find any significant changes in biomarker concentrations compared with baseline.^21^ Therefore, the detrimental effects documented in preclinical studies were not confirmed in the clinical setting in which an increase in pro-inflammatory cytokines likely occurs only in the initial stages after a PRP injection, which questions the clinical relevance in terms of the effect perceivable by the patient.

This trial with a high-level scientific design confirmed that leukocytes did not influence the clinical outcomes after PRP injections for the treatment of knee OA. These results are in contrast to those reported by previous meta-analyses that performed indirect comparisons between LR-PRP and LP-PRP, favoring the use of PRP without leukocytes.^1,18,27^ The direct comparison performed in the present RCT on 2 homogeneous groups led to a different conclusion, which is further strengthened by the use of a preparation method that allowed us to obtain 2 equivalent PRP formulations in terms of the platelet concentration but with a significant difference in terms of leukocytes, which remained the sole main distinguishing factor between the 2 PRP types. This allowed us to isolate the study variable (the role of leukocytes) and thus to clearly demonstrate its influence (or lack thereof) on the outcomes of the 2 study groups (LR-PRP and LP-PRP). A recent RCT conducted by Di Martino et al^13^ on 192 patients with knee OA already suggested that the presence of leukocytes in PRP does not affect its clinical efficacy. In fact, the authors reported no significant differences between freeze-thawed LR-PRP and freeze-thawed LP-PRP in terms of clinical outcomes, adverse events, and treatment failures. Nevertheless, that study focused on cryopreserved PRP, which remains a controversial topic. Freeze-thawing can impair platelet and leukocyte lifespan and function, alter the release pattern of growth factors, and favor the accumulation of pyrogenic cytokines.^20^ This limits the relevance of that study's findings when interpreting them in the clinical setting in which fresh PRP is commonly used. In this light, the current RCT overcomes this limitation by using fresh PRP, thus reducing the risk of alterations in PRP quality and clearly demonstrating the lack of influence of leukocytes on the clinical efficacy of PRP.

Safety is another important issue, in addition to efficacy, when using injectable products for the intra-articular treatment of knee OA. This study also demonstrated that leukocytes did not affect the safety profile of PRP injections, in contrast to what was previously highlighted in lower level studies hypothesizing a negative role in terms of adverse events.^19^ Filardo et al^14^ documented more frequent pain and swelling after LR-PRP injections compared with LP-PRP injections in 144 patients, although these events were minor and self-limiting and did not influence the final clinical results at 12-month follow-up, similar to our study. A recent RCT by Zhou et al^34^ recorded more mild adverse events (swelling and local pain) and worse early results in 30 patients treated with LR-PRP compared with 30 patients treated with LP-PRP, but the authors still reported similar efficacy over time. Moreover, Yaradilmis et al^32^ documented more transient local side effects after 3 injections of LR-PRP compared with LP-PRP in a prospective RCT on 90 patients with knee OA. Finally, Kim et al^19^ conducted a meta-analysis of 32 studies to compare clinical outcomes and adverse reaction rates between LP-PRP and LR-PRP for the treatment of knee OA, reporting a higher incidence of local symptoms with LR-PRP compared with LP-PRP, while no significant differences were found in the clinical outcomes. The current double-blind RCT overcomes the limitations of previous studies, which either had a lower number of patients and a weaker study design or indirectly evaluated leukocytes’ role in meta-analyses.^27^ Our direct comparison of 2 fresh PRP formulations, differing only in the leukocyte component, consolidates and strengthens the conclusion of the RCT by Di Martino et al^13^ on cryopreserved PRP, confirming that leukocytes did not have clinically relevant pro-inflammatory effects on the OA joint.

Leukocytes did not influence the clinical effects of PRP in this study, suggesting that the heterogeneous PRP effects documented in the literature may depend on a much more complex interplay of factors rather than the mere presence or absence of white blood cells. The interactions among the various components of PRP (platelets, leukocytes) could be crucial in influencing its effects, with a synergistic rather than individual cell concentration effect. Previous preclinical studies have investigated the use of various PRP formulations, reporting different responses based on different cell concentrations. Cavallo et al^10^ reported that PRP with a low concentration of platelets and few leukocytes resulted in greater cell growth and anabolism in terms of type II collagen and aggrecan production. On the other hand, PRP with high platelet and leukocyte concentrations showed a higher level of growth factors and cytokines, leading to higher hyaluronan production. Assirelli et al^2^ confirmed that PRP with a high concentration of platelets and leukocytes can upregulate pro-inflammatory factors while downregulating anticatabolic mediators. Conversely, PRP with a low concentration of platelets and without leukocytes did not demonstrate better results compared with simple platelet-poor plasma, thus suggesting that the lower platelet concentration in LP-PRP may result in the reduced secretion of bioactive molecules and consequently lower effects. This has been confirmed in the clinical setting, with a recent meta-analysis of RCTs demonstrating that PRP formulations with higher platelet concentrations provide superior pain relief and more durable functional improvement compared with PRP with low platelet concentrations in patients with knee OA.^5^ Future studies should directly compare the effects of different PRP formulations in terms of both leukocyte and platelet numbers, aiming to identify specific PRP types more suitable for treating patients with OA, while also considering the overall modest improvement documented in this study, which was reflected by the moderate percentages of patients reported as improved after the injection treatment.

Despite having high-level methodology, this study has some limitations. The randomization performed did not entirely prevent significant differences in baseline patient characteristics, with differences between the 2 groups in terms of age, EQ-VAS score, and knee ROM. These differences may introduce confounding bias, impacting the comparability of outcomes between the LP-PRP and LR-PRP groups. However, a comparison was performed on the improvement, which should reduce the possible influence of minor baseline differences. The LR-PRP group was slightly older, although these patients still belong to the same “middle age” category. Another limitation of this study is that the concentration of RBCs was different between the 2 groups, with higher values in the LR-PRP group. While it has been shown in vitro that RBCs may lead to significant cell death and the production of pro-inflammatory mediators, and their presence could have negatively affected the results of LR-PRP, we are still referring to very low concentrations.^8^ Moreover, in this light, the comparable findings of the 2 PRP types further confirm that LR-PRP did not lead to lower results than LP-PRP. Finally, imaging was performed to assess the level of OA before treatment, but the same imaging evaluations were not conducted at follow-up. However, imaging presents limitations in detecting differences in injection treatment studies because of the sensitivity of the procedure itself and limited OA progression at 12 months.^9^ Moreover, the primary goal of the study was to assess clinical outcomes and potential adverse events related to the presence of leukocytes. Despite these limitations, this study demonstrated that the presence of leukocytes did not influence the clinical outcomes of intra-articular PRP injections. Other factors should be investigated, with the aim to further optimize PRP injections for the treatment of patients with knee OA.

## Conclusion

This double-blind RCT demonstrated that leukocytes did not affect the safety and efficacy of intra-articular PRP injections for the treatment of patients with knee OA. Both LR-PRP and LP-PRP demonstrated comparable clinical outcomes at all follow-up time points, without showing differences in subjective and objective outcomes or in adverse events and treatment failures.
